# Electrical Property of Graphene and Its Application to Electrochemical Biosensing

**DOI:** 10.3390/nano9020297

**Published:** 2019-02-20

**Authors:** Jin-Ho Lee, Soo-Jeong Park, Jeong-Woo Choi

**Affiliations:** 1 Department of Chemical and Biomolecular Engineering, Sogang University, 35 Baekbeom-ro, Mapo-gu, Seoul 04107, Korea; jino@sogang.ac.kr; 2 Department of Chemistry and Chemical Biology, Rutgers, The State University of New Jersey, Piscataway, NJ 08854, USA; 3 Research Center for Disease Biophysics of Sogang-Harvard, Sogang University, 35 Baekbeom-ro, Mapo-gu, Seoul 04107, Korea; imation99@naver.com

**Keywords:** Graphene, Graphene Oxide, 2D materials, Electrochemical, Biosensor

## Abstract

Graphene, a single atom thick layer of two-dimensional closely packed honeycomb carbon lattice, and its derivatives have attracted much attention in the field of biomedical, due to its unique physicochemical properties. The valuable physicochemical properties, such as high surface area, excellent electrical conductivity, remarkable biocompatibility and ease of surface functionalization have shown great potentials in the applications of graphene-based bioelectronics devices, including electrochemical biosensors for biomarker analysis. In this review, we will provide a selective overview of recent advances on synthesis methods of graphene and its derivatives, as well as its application to electrochemical biosensor development. We believe the topics discussed here are useful, and able to provide a guideline in the development of novel graphene and on graphene-like 2-dimensional (2D) materials based biosensors in the future.

## 1. Introduction

Graphene, a single 2-dimensional (2D) layer of a hexagonal structure consisting of sp^2^ hybridized carbon atoms, and its derivatives have received increasing attention in biomedical fields, due to its unique physicochemical properties. This feature includes a high surface area, excellent electrical conductivity, strong mechanical strength, unparalleled thermal conductivity, and ease of surface functionalization (Table 1) [1,2,3,4,5].

Particularly, owing to the high surface area, excellent electrical conductivity, and capability to adsorb a variety of biomolecules, graphene has been considered as an ideal transducing material for constructing electrochemical biosensors [6,7]. It is well defined that the efficient electrochemical reaction takes place at the close distance between the electrode surface and the electroactive (reduction/oxidation, redox) site of a molecule. In detail, the electron transfer rate is inversely proportional to the exponential distance between the electrode surface and the electroactive redox site of the molecule [8,9]. Since the electron transfer between graphene and redox active molecule typically take place at either edge of the graphene layer or defects in the basal plane, the high surface area of 2D structure helps graphene to work as an excellent conducting material for electrical charge and heterogeneous electron transfer [10,11]. 

In addition, based on the unique atomic thin layer structure, the electrical properties of graphene are known to be highly sensitive to foreign atoms or absorbed molecules [12]. Favorably, graphene is known to be highly reliable for capturing aromatic molecules through a π–π stacking interaction. For example, single-stranded deoxyribonucleic acid (ssDNA) can bind to the graphene surface by π–π stacking interaction between deoxyribonucleic acid (DNA) and polyaromatic structures of graphene, and serve as a platform for various DNA based biosensing applications [13,14]. In addition, its derivatives graphene oxide (GO) are known to possess the oxygen-containing hydrophilic groups (hydroxyl and epoxy in the basal planes; carbonyl and carboxyl groups on the edges), that allows the electrostatic interaction [9,15]. Alternatively, graphene can be also functionalized by covalent bonding either through unsaturated p-bonds of graphene or oxygen-containing functional groups of GO [5,16]. As an example, various dienophiles, such as azomethine ylide, nitrene, and aryne, has been successfully generated a variety of terminal groups on the graphene surface for further modification [17,18,19]. The carboxyl groups of GO are used to link the amino groups of molecules by well-established carbodiimide chemistry [20]. According to the above mentioned unique physicochemical properties, the utilization of graphene as a functional component for an electrode has gained considerable interest in the field of electrochemical biosensors [19]. 

Although there exists an extensive collection of reviews for graphene synthesis methods and electrochemical sensing applications, the tremendous amount of recent activities and a new, live cell-based biosensing approach warrant a thorough review at this time. In this review, we will provide a selective overview of the recent advances on the synthesis methods of graphene and its derivatives, as well as its application in the electrochemical biosensor, which particularly covers small molecule, nucleic acid/protein, and live cell-based sensing. This review will provide an extensive analysis of the current state of the art and provide a perspective on key challenges that remain in the field. We hope that this review will inspire interest from various disciplines and highlight an important field wherein the advanced graphene-based electrochemical sensor is making great strides towards biomedical applications.

## 2. Synthesis of Graphene

**Figure 1:**
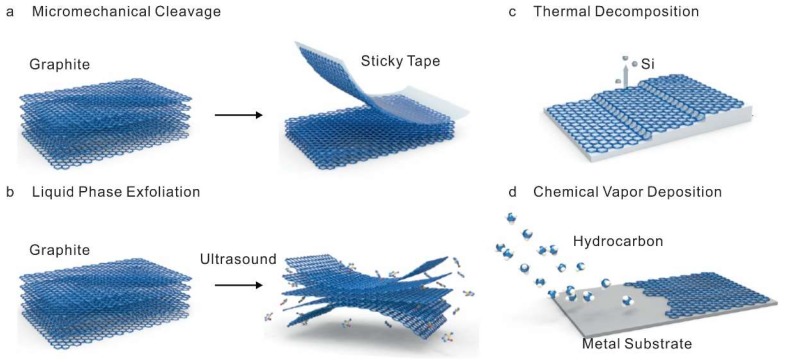
Schematic illustration of the graphene and its derivatives synthesis methods. (**a**) Mechanical exfoliation (**b**) liquid phase exfoliation, (**c**) thermal decomposition, and (**d**) chemical vapor deposition. Reproduced with permission from [23], Copyright Springer Nature, 2017.

As the outstanding physicochemical properties of graphene make this material promising candidate for electrochemical biosensor applications, the synthesis processes of the graphene which affect its properties also hold great influence on the proper development and performance of the biosensors. To this end, a number of a different synthesis method for graphene has been developed over the years. Particularly, in this review, most well-defined exfoliation phenomena or chemical vapor deposition (CVD) will be discussed as graphene and its derivatives synthesis methods (Figure 1) [9,21,22,23].

### 2.1. Mechanical and Chemical Exfoliation Method

The phenomena of exfoliation are to separate a few layers from the bulk material by overcoming the strong van der Waals attractions between adjacent layers. Among the exfoliation based process, the mechanical approach, ‘Scotch tape’ based exfoliation was first developed to obtain graphene, where few layers of graphene are peeled off from a highly ordered pyrolytic graphite (HOPG) flakes by external mechanical force (adhesive tape) based on the relatively weak interaction between the thin layers and the bulk materials. After the mechanical exfoliation, micromechanical cleavage of graphite, by rubbing a bulk crystal flake against another flake, was also utilized to obtain individual crystal planes of graphene layers [24]. In principle, through these mechanical exfoliation methods, high structural and electronic quality graphene crystals can be obtained without hosting structural defects into 2D graphene layers repeatedly [25,26,27]. However, these mechanical exfoliation methods are often limited for biosensor application, due to the difficulties in the mechanical cleavage of graphite crystals in a controlled manner. Such as low yield in the production of single-layer or few-layer graphene and relatively large lateral dimensions of graphene (range in micrometer size) often restrict their application in biosensors. Apart from the mechanical exfoliation, the potential energy caused by van der Waals attractions could be overcome in the presence of solvents as well [28]. This process, which is also known as liquid phase exfoliation (LPE), generally requires dispersion of bulk materials in a solvent, exfoliation, and purification [29]. Thus, the selection of ideal solvents is critical for LPE process to obtain high yield and stability [30]. However, the commonly used solvents for LPE, such as N,N-dimethylformamide (DMF) and N-methyl pyrrolidinone (NMP) are usually known to have acute toxic effects. To overcome this limitation, numerous approaches, such as urea-based aqueous exfoliation, which even showed higher efficiency compare to conventional DMF based exfoliation, were recently developed [31]. In addition, graphene derivatives can be also synthesized by the chemical oxidation of graphite. For example, one of most well established Hummer’s method for preparing graphite oxide includes the addition of potassium permanganate (KMnO4) to a mixture of graphite, sodium nitrate (NaNO3), and concentrated sulfuric acid (H_2_SO_4_) [32]. During the oxidation, small ions intercalate to bulk graphite oxide during oxidation and weaken the interlayer interactions. In detail, the sp^2^ hybridized carbon bonding is disrupted during the oxidation process, yielding formation of sp^3^ hybridized carbon bonding. Through this mechanism, sonication allows exfoliating GO layer from bulk graphite oxide flake and subsequent reduction of GO could also help to obtain reduce graphene oxide (rGO) [33,34,35]. Owing to ease of process, the exfoliation based on chemical method is known to be suitable for the synthesis of graphene at a large scale, which is important for the construction of bioelectronics devices, including electrochemical biosensors. In addition, the unique chemical structure of chemically derived GO and rGO, which differs from the pristine graphene or graphite, provide versatility in applications based on the chemical functionalization through the oxygen functional groups. However, large amounts of structural defects caused by inevitably introduced oxygen functional groups could affect the electrical properties of graphene and the performance of electrochemical biosensors. 

### 2.2. Thermal Decomposition and Chemical Vapor Deposition Method

An alternative approach to synthesize a high quality graphene layer in a large scale comprises the self-organization of carbon atoms on the surface of the crystal by the thermal decomposition of hydrocarbon and segregation of the carbon monolayer on metal substrates through CVD [36,37,38]. For example, the evaporation of silicon from single-crystalline silicon carbide (SiC) substrates at a high annealing temperature results in the organized attachment of the remaining carbon atoms on the surface of lattice-matched SiC substrates. Though, this method directly provides graphene layers on insulating SiC substrates in a wafer-scale; however, the strong interaction of graphene with substrates limits doping property, as well as transfer efficiency to other substrates for biomedical applications. Among the numerous metal substrates, nickel (Ni) and (copper) Cu substrates demonstrated the potential to separate graphene layer from the substrates and transfer onto other substrates, including a solid substrate to flexible and bendable substrates [36]. In detail, the synthesis mechanism for the metals substrate with high solubility of carbon species, such as Ni substrate include catalytic decomposition of the precursor, dissolution of decomposed carbon species, segregation of dissolved carbon atoms onto the metal surface, and followed by nucleation and growth of graphene layer on the surface of the substrate [39]. Though, the several critical factors, such as the thickness of Ni films, growth time, and cooling rate has been already revealed to improve the quality of synthesized graphene layers; however, it is still difficult to obtain a single layer of graphene through the polycrystalline Ni substrate and the electrical property of the synthesized graphene layer were found to be not very satisfactory as well. Comparably, by using a Cu substrate, which has low solubility of carbon, highly uniformed single graphene layer with the excellent electrical property was synthesized [36,40]. Due to the low solubility of carbon, the formation of graphene happens through the self-limited nucleation and lateral growth by diffusion of carbon atoms on the surface directly after the decomposition of precursors [41]. In addition, free-floating graphene layers could be obtained by metal etching or electrochemical bubbling method for further applications [42]. Although, CVD method can synthesize graphene layer even in a large area (up to several inches) with high electronic quality (mobility up to 10^5^ cm^2^V^−1^s^−1^) comparable to the exfoliation methods [36,39,43], the structural defects or contamination which can be originated during the etching and transfer process are still limiting factor for obtaining high profile graphene layer and can also affect the performance of electrochemical biosensors as well. 

## 3. Application to Electrochemical Sensing 

The biosensor is the analytical device which consists of a biological component that recognizes the target analytes and an electrical component (transducer) which converts the recognition event into a measurable signal. To improve the performance of biosensors, tremendous efforts from multidiscipline fields has been established. Ever since the discovery of graphene by Geim and Novoselov in 2004, numerous approach has been conducted to utilize graphene as transducing material to improve the performance of electrochemical sensors [6,44]. Graphene and its derivatives modified electrodes have exhibited excellent electrochemical behavior in terms of their high surface area and active electron transfer sites [7], which makes graphene as a promising electrode material to improve the performance of graphene and its derivatives based electrochemical biosensors. In this review, the division of biomedical electrochemical sensors will be divided into three categories i) small molecules, ii) nucleic acids and proteins, and iii) Live cell-based sensing.

### 3.1. Small Molecule Sensing

There are many small molecules that are highly relevant to human health and disease. Even a subtle change of these biomolecules could cause a serious disease which threatens a patient’s life [45,46,47,48]. For example, dopamine (DA) is one of the most important neurotransmitters that play a vital role in the central nervous system. Abnormal level of DA can cause severe neurological disorders, such as Parkinson’s disease [49,50,51]. However, due to the complex matrices of nature, it is still challenging to develop a biosensor to distinguish the biomolecules which share a similar oxidation potential, such as ascorbic acid (AA), uric acid (UA) and other catecholamine molecules. To resolve this problem, graphene has been adopted as a transducing material in the development of the electrode. Through its phenyl structure, these molecules could adsorb on the graphene-modified electrode surface through the different π-π stacking interactions. Ping et al. introduced a graphene-based screen-printing ink which could selectively and sensitively analyze these molecules via differential pulse voltammetry (DPV) [52]. Even in the co-existence of these three molecules, the linear range and detection limit were found to be 0.5–2000 μM, 4.0–4500 μM, and 0.8–2500 μM and 0.12 μM, 0.95 μM, and 0.20 μM for DA, AA, and UA, respectively. 

**Figure 2:**
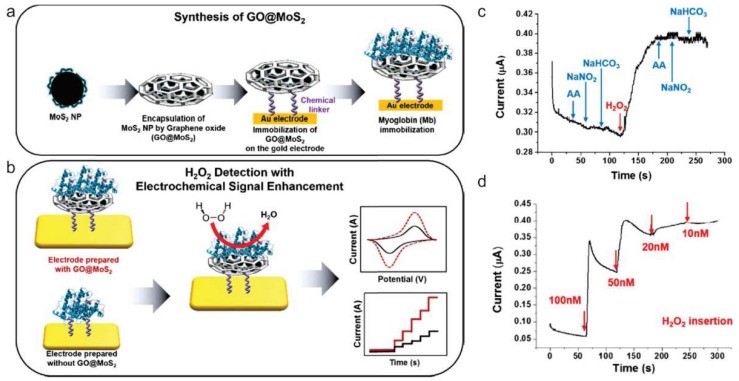
(**a**) Schematic of graphene oxide encapsulated molybdenum disulfide (MoS2) nanoparticle preparation for the fabrication of electrochemical biosensors composed of myoglobin (Mb) and (**b**) its application to H_2_O_2_ detection with improved electrochemical performance. (**c**–**d**) Amperometric response curves obtained from Mb/GO@MoS_2_ upon successive (**c**) addition of 100 nM L-ascorbic acid (AA), 100 nM sodium nitrite (NaNO_2_), 100 nM sodium bicarbonate (NaHCO_3_), and 100 nM H_2_O_2_ solutions; and by (**d**) addition of 100, 50, 20, and 10 nM H_2_O_2_ solutions. Reproduced with permission from [56], Copyright Elsevier, 2017.

As an alternative approach, graphene was also utilized to provide large surface area, as well as a binding motif for the mediator (i.e., enzyme) to improve the electrocatalytic performance of electrochemical sensors to determine small molecules, such as hydrogen peroxide (H_2_O_2_), glucose, and nicotinamide adenine dinucleotide (NADH) sensitively. Besides its well-known cytotoxic effects, as an essential mediator in many biological processes, H_2_O_2_ detection has earned great attention. However, due to the co-existing other electro-active constituents, the detection of H_2_O_2_ is easily interfered [53,54]. To improve the H_2_O_2_ detection efficiency, graphene was also utilized to improve the performance of electrochemical sensors. Fan et al., designed a graphene capsule, which served as a carrier for horseradish peroxidase (HRP), to detect H_2_O_2_ in human serum [53]. Through the large surface area and high conductivity of graphene, the synergistic effect on catalytic activity was able to be obtained. Instead of using the enzyme, Wang et al., have grown Prussian blue nanocubes on the surface of nitrobenzene-functionalized reduced graphene oxide as an “artificial enzyme peroxidase” for constructing H_2_O_2_ electrochemical biosensors [55]. Another approach based on the incorporation of myoglobin (Mb) on graphene oxide encapsulated molybdenum disulfide (MoS_2_) nanoparticle (Mb-GO@MoS_2_) hybrid structure was also reported by Choi’s group (Figure 2) [56]. Mb is a one of metalloprotein family with unique redox properties, due to the metal ion core integrated into the hemin group. Through this unique redox properties, Mb can be used to detect H_2_O_2_ through electrochemical reduction of H_2_O_2_ as well. The developed Mb-GO@MoS_2_ structure and extended electroactive surface also affected the fast electron transfer and resulted in an enhanced amperometric response H_2_O_2_. Instead of using full protein, Song et al. utilized hemin porphyrin and functionalized on the graphene/GNP/glassy carbon electrode to avoid the possible insulating effect from protein structure [57]. Note that hemin porphyrin is a well-known natural metalloporphyrin which is the active site in heme-proteins, such as hemoglobins and myoglobins [58]. Alternatively, Shao et al. have shown that by just doping graphene nitrogen, the better electrocatalytic activity could be obtained for H_2_O_2_ detection as well [59]. The enhanced performance of nitrogen-doped graphene is expected, due to the existence of nitrogen functional groups in addition to oxygen-containing groups and structural defects. In a similar manner, graphene was also utilized to improve the electrocatalytic performance of electrochemical sensors to determine other small molecules, such as DA, glucose, and NADH as well. The recent researches on graphene-based electrochemical biosensors toward various small molecules are compared in Table 2.

### 3.2. Nucleic Acid and Protein Sensing

**Figure 3:**
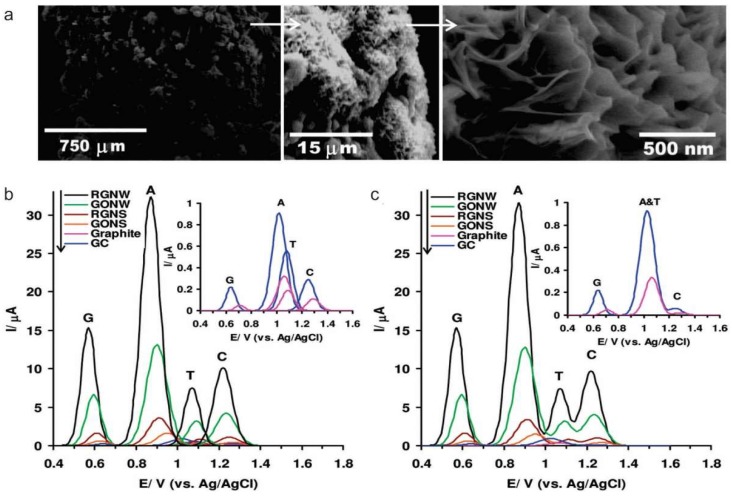
(**a**) Scanning electron microscopy images of the graphene oxide nanowalls deposited on a graphite rod by using electrophoretic deposition (**b**,**c**) Differential pulse voltammetric profiles of the reduced graphene nanowalls (RGNW), graphene oxide nanowalls (GONW), reduced graphene nanosheet (RGNS), and graphene oxide nanosheet (GONS) electrodes as compared to the graphite and glassy carbon (GC) electrodes for detection of (**b**) the four free bases of DNA (G, A, T, and C) separately, and (**c**) equimolar mixture of G, A, T, and C. Reproduced with permission from [71], Copyright American Chemical Society, 2012.

A sensitive and selective nucleic acids (DNA/ribonucleic acid, RNA) sensor is in high demand for the diagnosing gene-related diseases. DNA sensors also referred to as geno-sensors, are an analytical system, which integrates a sequence-specific probe on a transducer. Thus, the immobilization of DNA strands greatly influences the performance of the electrochemical DNA sensor. In this manner, graphene and its derivatives provide an excellent avenue to develop electrochemical DNA sensor. Hu et al. utilized GO as a DNA probe immobilization layer for electrochemical detection of HIV-1 gene fragment [70]. First, GO is anchored on diazonium functionalized electrode surface via electrostatic attraction, hydrogen bonding or epoxy ring opening. The π-π stacking interaction between the aromatic ring of GO and DNA base ring facilitated DNA immobilization, and impedance measurement was used for the quantitative detection of HIV-1 gene fragment up to 0.11 pM. Moreover, Akhavan et al. developed a graphene nanowall structure and showed an extremely high response to single-strain DNA towards single-strain DNA electrochemical sensing. As a result, they have observed a unique response signal from each kind of basic group through DPV measurement (Figure 3) [71]. 

**Figure 4:**
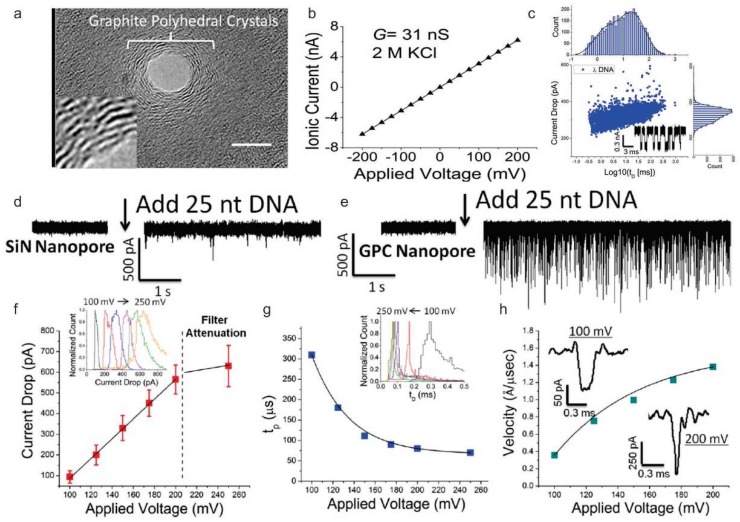
(**a**) Transmission electron microscopy image of the shrunked nanopore with graphite polyhedral crystal (GPC)-edges sculpted by the irradiation of electron beam (6.2 × 10^5^ electrons/nm^2^·s) on to the single layer graphene. Scale bar = 5 nm. (**b**) Current versus voltage plot for a 5 nm nanopore (in diameter) having GPC-edges in the 2M KCl condition. (**c**) Current drop translocation time scatter plot for double stranded λ-DNA (5 nM concentration, 48.5 kb long) using a 5 nm nanopore with a GPC-edges at 250 mV [1 M KCl, 10 mM Tris, 1 M ethylenediaminetetraacetic acid (EDTA)]. The mean current drop value was 332 ± 62 pA by the scatter plot. (**d**–**e**) Ionic current traces for single-stranded DNA (25 bases in length) in (**d**) a 5 nm silicon nitride nanopores (50 nm thick) and (**e**) 5 nm graphene nanopores with GPC-edges. (**f**–**h**) Detailed characterization of 5 nm graphene nanopores with GPC-edges for 25 nucleotide-long DNA fragment sensing. (**f**) The linear increase in current drop based on applied voltages, (**g**) The exponential decrease of peak translocation time based on applied voltages, and (**h**) Calculated translocation velocity from (**e**). The velocity of the 25 nucleotide-long DNA fragment in 5 nm graphene nanopores with GPC-edges was 0.35 Å/μs at 100 mV/room temperature, which demonstrates the slower velocity than the silicon nitride pores. Events were recorded at 100, 125, 150, 175, 200, and 250 mV in 2 M KCl, 10 mM Tris (pH 8), 1 mM EDTA and 10 nM DNA at room temperature. Reproduced from Reference [74] with permission from the American Chemical Society.

Although it is clear that nucleic acids can effectively immobilize on the graphene and its derivatives, many researchers have focused to modify the graphene and its derivatives electrode surface with various materials to achieve improved performance on electrochemical sensor. For example, Tiwari et al. electrophoretically deposited graphene oxide modified iron oxide-chitosan hybrid nanocomposite onto indium tin oxide (ITO) coated glass substrate and utilized for the detection of a pathogenic Escherichia coli DNA with a detection limit of 10 fM [72]. In addition to the surface modification, the enzyme was also utilized to improve electrochemical sensor. Esteban-Fernández de Ávila et al. designed a disposable electrochemical DNA sensor based on carboxymethyl-cellulose-rGO modified screen-printed carbon electrodes. And HRP was utilized to catalyze the redox mediator, tetramethylbenzidine (TMB), and the substrate (H_2_O_2_) for the detection of the p53 tumor suppressor gene [73]. Besides, graphene can be also utilized for DNA sequencing. With the presence of nanopore on the monolayer graphene, detailed electric signals can be sensed when DNA passes the pore by measuring transverse conductance of DNA. Through this mechanism, Freedman et al. have distinguished long and short DNA using nanopores with graphite polyhedral crystal (GPC)-edges (Figure 4) [74].

**Figure 5:**
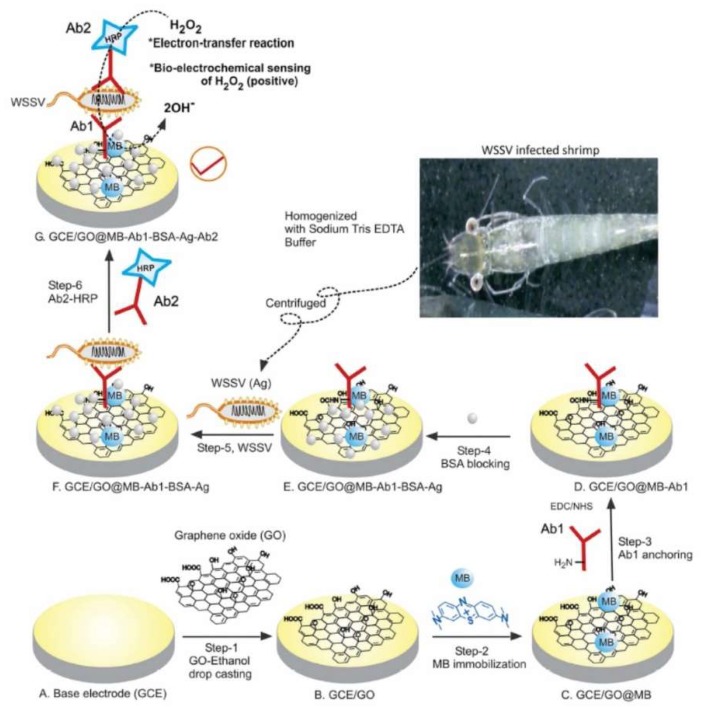
Schematic illustration for the development of electrochemical white spot syndrome virus immunosensor using a methylene blue dye (MB) immobilized graphene oxide modified glassy carbon electrode. Reproduced with permission from [76], Copyright Springer Nature, 2017.

In parallel, many biological processes can be also monitored by quantification of specific proteins. Owing to the amphiphilic nature of graphene and its derivate, it provides sufficient active sites to immobilize these probes, including aptamer and antibody to detect specific proteins as well. Wen et al. explored hairpin-shaped DNA aptamer as a cognition element for carcinoembryonic antigen on the gold nanorods functionalized graphene electrode surface [75]. In addition, by targeting membrane protein of pathogenic microbes, Natarajan et al. successfully developed an immunoassay for white spot syndrome virus using a methylene blue dye (MB) immobilized graphene oxide modified glassy carbon electrode (GCE/GO@MB) (Figure 5) [76]. Here, graphene was also utilized to improve antibody immobilization efficiency and enhance electron transfer, the binding on the target produced an enhanced immune-recognition response by the sandwich assay with an enzyme reaction. As mentioned above, considerable approaches have been made to develop and improve graphene-based electrochemical DNA and protein sensors. However, the simultaneous detection of multiple targets in complex biological matrices still remains a major bottleneck for clinical analysis. The recent researches on graphene-based electrochemical biosensors toward various protein and nucleic acids are compared in Table 3.

### 3.3. Live Cell-based Sensing

**Figure 6:**
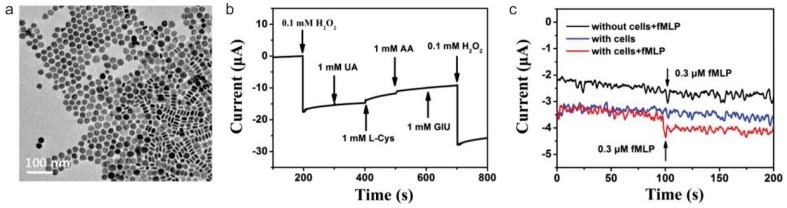
(**a**) Transmission electron microscopy image of PtPb nanoplates. (**b**) Chronoamperometric curves of the graphene/Intermetallic PtPb nanoplates composite (PtPb/G) electrode with the successive addition of 0.1 mM H_2_O_2_, 1 mM Uric Acid (UA), 1 mM L-Cysteine, 1 mM ascorbic acid (AA), and 1 mM glucose at a constant potential at −0.2 V. (**c**) Amperometric responses of the PtPb/G electrode to the addition of N-formyl methionyl-leucyl-phenylalanine (fMLP) with and without Raw 264.7 cells. Reproduced with permission from [85], Copyright American Chemical Society, 2017.

Owing to their excellent biocompatibility, solubility, and unique interactions with specific molecules, graphene, and its derivatives has been also utilized to detect a response from the biological process of living cells. For example, effective and accurate characterization of H_2_O_2_ concentration in a living cell is critical to achieving the normal physiological activities of cells. Wu et al. integrated nitrogen-doped graphene to monitor H_2_O_2_ release process from live cells through improved electrocatalytic activity [84]. After the injection of phorbol 12-myristate-13-acetate to induce H_2_O_2_ generation in the neutrophil cells, the rapid increase of amperometric response was able to be observed, which indicates a large amount of H_2_O_2_ release from the cells. Sun et al., also reported a graphene/Intermetallic platinum/lead (Pt/Pb) nanoplates composites for sensing H_2_O_2_ release from live macrophage cells (Raw 264.7) (Figure 6) [85]. Through the high-density of electrocatalytic active sites on the unique PtPb nanoplates and the synergistic effect with graphene contributed for outstanding electroanalytical performance. The proposed construct showed 12.7 times higher redox signals than that of commercial Pt/Carbon electrode and able to detect H_2_O_2_ with a wide linear detection range of 2 nM to 2.5 mM. Zhang et al. also proposed a way to monitor H_2_O_2_ secretion from viable cells with a freestanding nanohybrid paper electrode composed of 3D ionic liquid (IL) functionalized graphene framework (GF) decorated by gold nanoflowers [86]. The gold nanoflower modified IL–GF was synthesized by a dopamine-assisted one-pot self-assembly method. The resultant nanohybrid paper electrode exhibits good non-enzymatic electrochemical sensing performance toward H_2_O_2_. Through the real-time tracking of H_2_O_2_ release from different breast cells attached to the paper electrode allow to distinguish the normal breast cell line HBL-100 from the cancer breast cell line MDA-MB-231 and MCF-7 cells. Liu et al. utilized HRP on a porous graphene electrode to monitor the H_2_O_2_ release from living cells [87]. A simple method based on silver nanoparticles etching process was proposed to prepare porous graphene network. Owing to the versatile porous structure, the analysis performance was significantly improved by loading large amounts of enzyme and accelerating diffusion rate. A significant low detection limit of 0.0267 nM and wider linear range of 7 orders of magnitude were achieved. A rat adrenal medulla pheochromocytoma cell line PC12 was chosen as a model cancer cell, and H_2_O_2_ release was monitored within AA stimulation. In a similar manner, Li et al. monitored nitric oxide (NO) by developing a new 3D hydrogel composite via in situ reductions of Au^3+^ on three-dimensional graphene hydrogel [88]. The developed sensor showed improved electrochemical performance compare to pure gold nanoparticles, pure graphene, 3D graphene hydrogels, and gold nanoparticle-graphene hybrids. A linear relation was obtained for 0.05–0.4 mM of NO. Two different normal and cancer skin cell was stimulated with Ach, and concentration-dependent signal increments were analyzed. 

**Figure 7:**
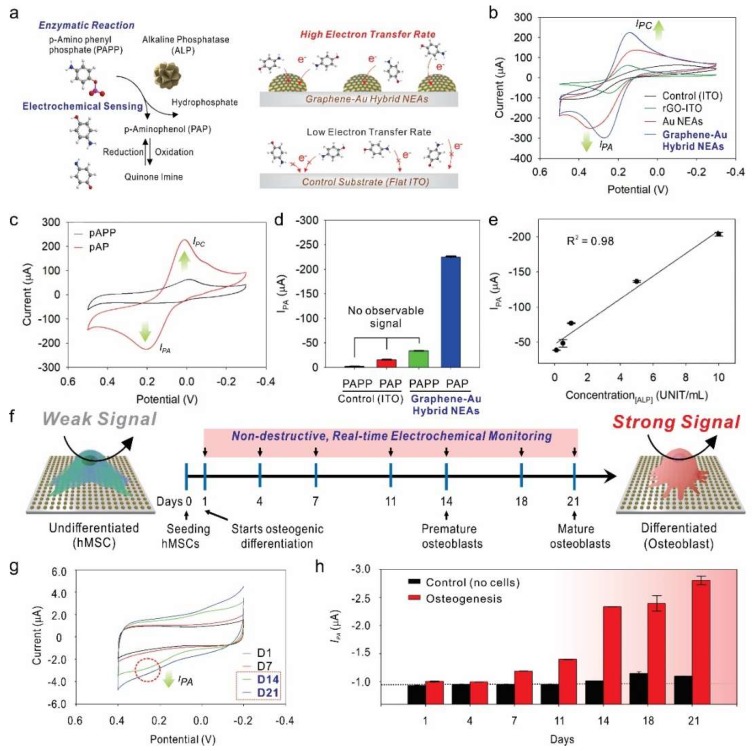
(**a**) Schematic illustration of alkaline phosphatase (ALP) based enzymatic reaction and electrochemical sensing mechanism on the 3D surface in graphene-Au hybrid nanoelectrode arrays (NEAs) compared to 2D flat ITO surface. (**b**) Improved voltammetric response of graphene-Au NEAs compares to bare ITO substrate, rGO-coated ITO substrate, and Au NEAs. (**c**,**d**) Cyclic voltammogram and Anodic peak (oxidation potential: *I*_PA_) value of P-aminophenyl phosphate (PAPP) on graphene-Au NEAs before and after enzyme reaction with ALP. (**e**) The linear correlations between concentrations of ALP and the current signal at *I*_PA_. (**f**) Schematic illustration of electrochemical signal change between undifferentiated hMSCs and differentiated osteocyte based on ALP generation. (**g**) Cyclic voltammetry, and (**h**) calculated *I*_PC_ values from time-dependent monitoring during osteogenesis of hMSCs (range from D1 to D21). Reproduced with permission from [89], Copyright John Wiley and Sons.

Differently, Lee et al., utilized graphene-Au hybrid nanoelectrode array (NEAs) to monitor stem differentiation in a non-destructive real-time manner (Figure 7) [89]. Typically, unique multifunctional graphene-Au hybrid NEAs were fabricated via laser interference lithography and physical vapor deposition methods. Followed by surface modification with reduced graphene oxide. The presence of reduced graphene oxide enhanced the cell adhesion and spreading without functionalization with any extracellular matrix proteins, which could work as an insulator and diminish ET between the electrode and electroactive molecules. Owing to the excellent biocompatibility and electrochemical performance of graphene-Au hybrid NEAs, the osteogenic differentiation of human mesenchymal stem cell was successfully monitored through an alkaline phosphatase (ALP)-based enzymatic reaction. During the osteogenesis, ALP expression level is known to be sequentially increased. P-aminophenyl phosphate (PAPP) were introduced to cell prior to electrochemical monitoring, the ALP expressed on the cell catalytically hydrolyzed the PAPP to produce electroactive p-aminophenol (PAP), and the redox reaction between PAP and Quinone imine (QI) was monitored by cyclic voltammogram. Through this mechanism, the osteogenic differentiation of human mesenchymal stem cell was successfully monitored in both non-destructive and real-time manner. Although, stem cell therapy has arisen as a promising method in the field of biomedicine owing to their unique ability to differentiate into multiple cell lineages [90], necessary required destructive analysis process, such as cell lysis and cell fixation were one of a critical limiting factor for further clinical applications. Such a novel electrochemical detection method proposed by graphene-Au hybrid NEAs could be a breakthrough in the preclinical investigation of differentiated stem cells. Consequently, this kind of work is expected to be highly potential to advance stem cell differentiation assays by providing a practical, non-destructive, real-time monitoring tool. The recent researches on graphene-based electrochemical biosensors toward various live cell-based sensing strategies are compared in Table 4.

## 4. Conclusions and Future Outlook

It is evident that the exceptional physicochemical properties of graphene and its derivatives make them compelling for various electrochemical biosensor applications. The development and wide application of the electrochemical sensing based on these materials were hindered by the lack of facile and reproducible synthesis method of these materials with defined properties. In order to make it compatible to develop various electrochemical sensors, numerous efforts have been devoted to controllable and scalable production of graphene and its derivatives. Mechanical exfoliation method was first used to obtain graphene; however, the poor controllability and low production were the limiting factors. To achieve scalable production, LPE based methods were developed to obtain a dispersed solution of these materials and its products has been the most widely utilized for the electrochemical biosensors. More or less, recently CVD method was also adopted to synthesize graphene with controllable properties; however, limited scale production of graphene and its derivatives still hinders its applications. For example, various oxygenated surface functional groups provide a relatively high surface area, while it inhibits the performance of electrochemical biosensor as an insulator. Thus, a novel synthesis method which can consistently produce graphene and its derivatives with defined properties (high quality) in a large scale (high yield) and cost-effective manner is still required for future commercialization of graphene and its derivatives based electrochemical biosensors.

During the past years, many different strategies have been also explored to develop novel graphene and its derivatives based electrochemical biosensors for analyzing bio/chemical molecules. It is clear that the optimization through the selection of a suitable surface functionalization method still needs to be revealed, in order to develop a highly effective and reproducible electrochemical biosensors. In addition, the combination with functional materials, such as ionic liquids, nanomaterials and polymers, have provided numerous choices to take the synergistic effect to enhance the electroanalytical performances. However, designing and finding the appropriate combinatorial structure with functional materials must be addressed for each biomolecule to maximize the performance of electrochemical biosensors. Although extensive studies have been made to design and fabricate novel electrochemical biosensors, long-term stability of combinatorial structure and functionalized surfaces (with receptor), in complexed real sample matrices, should be considered. Real biological fluids, such as blood and plasma, always contain various molecules and ions which can cause the interference through nonspecific binding events and undermine the performance of electrochemical biosensors. Furthermore, to adopt graphene and its derivatives for recently arisen in situ live cells and in vivo sensing strategies, more effort, such as long-term toxicity of graphene and its derivatives, as well as incorporated functional materials, should be also discovered. Moreover, since graphene and its derivatives can serve as both the sensing component and transducer, utilization to flexible electrode and miniaturization in size by forming free-standing structures via self-assembly would be an excellent approach for developing an implantable (flexible) and portable (lightweight) sensors as well. However, the lack of a suitable power source is a limiting factor.

In addition, owing to the advantages of specific planar morphology, graphene-like 2D nanomaterials have attracted significant attention as emerging materials for electrochemical sensor approaches. Taking the advantages of diverse composition and structural effect, graphene-like 2D nanomaterials also have promoted great efforts to improve the performance of electrochemical biosensors. Similar to graphene and its derivatives, the defined synthesis method for high quality graphene-like 2D nanomaterials with controllable sizes and thickness, as well as tunable properties, and surface functionalization method is highly desirable for their practical application to electrochemical biosensor development as well. In witness of their current attention, we also endeavor that further improvement of graphene and its derivatives, as well as graphene-like 2D nanomaterial-based electrochemical biosensors, will lead to a significant advance in analytic applications for the highly effective and reliable detection of biomarker and open new avenues in biological and medical fields.

## Figures and Tables

**Table 1:** Physicochemical properties of graphene and its derivatives.

Physicochemical Property	Estimated Value	Ref.
High surface area	~2630 m^2^g^−1^	[1]
Excellent electrical conductivity	~1738 siemens/m	[2]
Strong mechanical strength	Young’ Modulus ~1100 GPa, Fracture strength ~125 GPa	[3]
Thermal conductivity	5000 Wm^−1^K^−1^	[4]
Ease of functionalization	π–π stacking interaction Electrostatic interaction	[5]

**Table 2:** Comparison of different graphene-based electrode for small molecule detection.

Electrode Materials	Target	Linear Range	Detection Limit	Ref.
Graphene capsule/horseradish peroxidase	H_2_O_2_	0.01–12 mM	3.3 μM	[53]
Prussian blue nanocubes/nitrobenzene/reduced graphene oxide	H_2_O_2_	1.2 μM–15.25 mM	0.4 μM	[55]
Myoglobin (Mb)/MoS2 nanoparticle/graphene oxide	H_2_O_2_	-	20 nM	[56]
Hemin porphyrin/graphene/gold nanoparticle	H_2_O_2_	0.3 μM–1.8 mM	0.11 μM	[57]
Cobalt ferrite nanoparticles decorated exfoliated graphene oxide	H_2_O_2_ NADH	0.9–900 μM 0.50–100 μM	0.54 μM 0.38 μM	[60]
Au-Ag nanoparticles/poly(L-Cysteine)/reduced graphene oxide	NADH ethanol	0.083 µM–1.05 mM 0.017 µM–1.845 mM	9.0 nM 5.0 µM	[61]
Graphene-pyrroloquinoline quinone	NADH	0.32 µM–220 µM	0.16 µM	[62]
FeN nanoparticles/nitrogen-doped graphene core-shell	NADH	0.4 µM–718 μM	25 nM	[63]
				
Screen-printed graphene	Dopamine Ascorbic acid Uric acid	0.5 µM–2000 μM 4.0 µM–4500 μM 0.8 µM–2500 μM	0.12 μM 0.95 μM 0.20 μM	[52]
Nickel and copper oxides-decorated graphene	Dopamine	0.5 µM–20 μM	0.17 μM	[64]
Molecularly imprinted polymer modified graphene/carbon nanotube	Dopamine	2.0 fM–1.0 pM	667 aM	[65]
Gold nanoparticle-anchored nitrogen-doped graphene	Dopamine glucose	30 nM–48 μM 40 μM–16.1 mM	10 nM 12 μM	[66]
Graphene-encapsulated gold nanoparticle	glucose	6 μM–28.5 mM	1 μM	[67]
Cobalt phthalocyanine–ionic liquid–graphene	glucose	0.01–1.3 mM and 1.3–5.0 mM	0.67 µM	[68]
Copper nanoparticle/graphene oxide/single wall carbon nanotube	glucose	1 μM–4.538 mM	0.34 μM	[69]

**Table 3:** Comparison of different graphene-based electrode for protein/nucleic acid detection.

Electrode Materials	Target	Linear Range	Detection Limit	Ref.
Graphene Oxide/probe DNA	HIV-1 gene (cDNA)	1 pM–1 μM	0.11 pM	[70]
Reduced graphene nanowalls	dsDNA	0.1 fM–10 mM	9.4 zM	[71]
Graphene oxide modified iron Oxide/chitosan/probe DNA	*Escherichia coli* Ο157:¨Η7 gene (cDNA)	10 fM–1 μM	10 fM	[72]
Screen-printed carbon/reduced graphene oxide/Carboxy-methyl-cellulose/probe DNA	p53 tumor suppressor gene (cDNA)	10 nM–0.1 μM	2.9 nM	[73]
Nitrogen-doped graphene/Au nanoparticles/probe DNA	multidrug resistance gene	10 fM–100 nM	3.12 fM	[77]
Fe3O4 Nanoparticles/reduced graphene oxide	HIV-1 gene (cDNA)	10 aM–100 pM	-	[78]
Glassy carbon/reduced graphene oxide/polypyrrole–3–carboxylic acid	Breast cancer 1 gene	1 pM–0.1 μM	0.3 pM	[79]
Gold nanorods/graphene/ hairpin-shaped DNA aptamer	Carcinoembryonic antigen	5 pg·mL^−1^–50 ng·mL^−1^	1.5 pg·mL^−1^	[75]
Graphene quantum dot-ionic liquid-nafion/hairpin aptamer	Carcinoembryonic antigen	0.5 fg·mL^−1^–0.5 ng mL^−1^	0.34 fg·mL^−1^	[80]
Graphene/glassy carbon/aptamer	Carcinoembryonic antigen	80 ag·mL^−1^–950 fg·mL^−1^	80 ag·mL^−1^	[81]
Glassy carbon/graphene oxide methylene blue/Antibody	White spot syndrome virus	1.36 × 10^−3^–10^7^ copies·µL^−1^	10^3^ copies·µL^−1^	[76]
Graphene-wrapped copper oxide/cysteine	*E. coli* O157:H7	10 CFU·mL^−1^–10^8^ CFU·mL^−1^	3.8 CFU·mL^−1^	[82]
Gold/reduced graphene oxide/polyethylenimine	*E. coli*	10 CFU·mL^−1^–10^4^ CFU·mL^−1^	10 CFU·mL^−1^	[83]

**Table 4:** Comparison of different graphene-based electrode for live cell-based detection.

Electrode Materials	Target	Linear Range	Detection Limit	Ref.
Nitrogen doped graphene	H_2_O_2_	0.5 μM–1.2 mM	0.05 μM	[84]
Graphene/PtPb-nanoplate	H_2_O_2_	2 nM–2516 μM	2 nM	[85]
Gold nanoflowers modified ionic liquid functionalized graphene framework	H_2_O_2_	0.5 μM–2.3 mM	100 nM	[86]
HRP supported Porous graphene	H_2_O_2_	2.77 μM –835 μM	26.7 pM	[87]
Graphene-Pt nanocomposites	H_2_O_2_	0.5 μM–0.475 mM	0.2 μM	[91]
GNP deposited 3D graphene hydrogel	NO	200 nM –6 μM	9 nM	[88]
GNP/calf thymus DNA/nitrogen-doped graphene	NO	2 nM–500 nM	0.8 nM	[92]
Iron phthalocyanine decorated nitrogen-doped graphene on ITO	NO	0.18 μM–400 μM	0.18 μM	[93]
3-aminophenylboronic acid functionalized graphene foam network	H_2_S	0.2 μM–10 μM	50 nM	[94]
Dendritic Pt nanoparticles decorated freestanding graphene paper	DA	87 nM–100 μM	5 nM	[95]
Zn-NiAl layered double hydroxide on reduced graphene oxide	DA	1 nM–1 μM	0.1 nM	[96]
Aryldiazonium Salts and GNP decorated reduced graphene oxide	TNF-α	0.1–150 pg·mL^−1^	0.1 pg·mL^−1^	[97]
Graphene-Au hybrid nanoelectrode array	ALP	0.1–10 unit·mL^−1^	0.03 unit·mL^−1^	[89]
